# Income inequality and socioeconomic differences in bullying perpetration among adolescents in post-communist countries of Europe: Findings from the HBSC study

**DOI:** 10.1016/j.pmedr.2023.102540

**Published:** 2023-12-09

**Authors:** Armen Albert Torchyan, Inge Houkes, Hans Bosma

**Affiliations:** a Department of Social Medicine, Care and Public Health Research Institute (CAPHRI), Faculty of Health, Medicine and Life Sciences, Maastricht University, P.O. Box 616, 6200, MD, Maastricht, The Netherlands; b Department of Family and Community Medicine, College of Medicine and King Khalid University Hospital, King Saud University, P.O. Box, 7805, Riyadh 11472, Saudi Arabia

**Keywords:** Adolescent, Bullying, Golden youth, HBSC, Income inequality, Neoliberal, Post-Communist countries of Europe, Socioeconomic status

## Abstract

**Background:**

Adolescent bullying is a major public health concern in post-Communist countries of Europe (PCCE). We studied the impact of income inequality on the prevalence of and socioeconomic differences in bullying perpetration.

**Methods:**

Nationally representative samples of adolescents aged 11–15 years from 14 PCCE participating in the Health Behavior in School-aged Children survey during 2017–18 years were included. Multilevel logistic regression models were fitted.

**Results:**

Overall, 71,119 adolescents participated in the survey. About 27.7 % of country differences in bullying perpetration were attributable to high income inequality. Adolescents with low and high family socioeconomic status (SES) had a 10–15 % greater risk (*P* < 0.05) of bullying perpetration compared to middle-SES adolescents. In cross-level interaction, the risk of bullying perpetration was the highest among high-SES adolescents from countries with wider income inequality (*P*-interaction = 0.003). The odds ratios of bullying perpetration comparing high- vs. low-SES adolescents ranged from 0.67 in the least unequal to 1.59 in the most unequal countries.

**Conclusions:**

Our findings suggest that high income inequality might contribute to school bullying among adolescents in PCCE, particularly those from high-SES families. The necessary political and economic interventions should focus on improving the country socioeconomic environment in PCCE. Moving towards less neoliberal policies and creating a strong welfare state that promotes the population's economic and social well-being and reduces income inequality might be a necessary component of macro-level strategies aimed at preventing bullying among adolescents. Targeted public health interventions might be required for high-SES adolescents in countries with wide income inequality.

## Highlights

• Adolescent bullying is a major public health concern in post-Communist Europe.
• High income inequality might contribute to school bullying in post-Communist Europe.
• Wider income inequality can incite high-SEP adolescents to bullying perpetration.
• Affluent youth might need public health interventions in the most unequal countries.
• Less neoliberal policies and a strong welfare state might be the remedy.

## 1 Introduction

A safe and supportive environment is essential for healthy development and a secure future (Black et al., 2017). Yet, bullying is a major public health concern in post-Communist countries of Europe (PCCE). It has been estimated that bullying victimization affects 28.9 % of adolescents aged 15 years in PCCE. In comparison, it is a third more than in the rest of Europe and 2.4 times greater than in the Netherlands (Hosozawa et al., 2021). Substantial country differences in bullying perpetration (Hosozawa et al., 2021) suggest that more upstream, macro-level social, political, and economic mechanisms might contribute to bullying behavior among adolescents and generate inequalities therein. The latter is supported by the socio-ecological model of violence (Krug et al., 2002).

After the Cold War, many PCCE widely adopted neoliberal reforms to attract business investments and recover the ruined economy (Appel and Orenstein, 2016). To some extent, neoliberal policies aimed at strengthening the private sector's role in the economy and reducing public social expenditures helped improve their economies. However, long-lasting and even radical neoliberal transformations in many PCCE widened the material consequences of neoliberal policies and the gap between rich and poor, aggravating income inequality (Appel and Orenstein, 2016). In addition, neoliberal policies affected various social institutions (Appel and Orenstein, 2016), which are supposed to decrease income inequality, buffer its negative effects, and protect the rights of individuals from less advantaged socioeconomic backgrounds (Coburn, 2004).

Wilkinson (Wilkinson and Pickett, 2011) suggested that above a certain level of a country economic development, about USD 5,000 of gross domestic product (GDP) per capita, further improvement in the economy has relatively little effect on population health. Instead, increased income inequality can cause higher rates of health and social problems. Thus, he contends that in countries with large income inequality, people are more concerned with their social standing and more vigilant about losing their status, causing increased status anxiety. Similarly, Wilkinson (Wilkinson and Pickett, 2011) claims that school bullying might be more common in countries with greater income inequality, where adolescents might resort to bullying to maintain or achieve a higher status in the peer hierarchy.

In this paper, we utilized nationally representative data (The HBSC Data Management Centre, 2017) on 14 PCCE with GDP per capita of more than USD 5,000, participating in the Health Behavior in School-aged Children (HBSC) study. We used the Gini index for country income inequality to examine the extent to which country differences in bullying perpetration are attributable to high income inequality. In addition, we examined the impact of income inequality as an upstream contextual factor on individual-level socioeconomic differences in bullying perpetration, which might help to identify potential solutions for preventing this harmful behavior.

## 2 Methods

### 2.1 Study design

The HBSC study is a nationally representative school-based survey conducted across Europe and North America between 2017 and 2018, employing a standardized methodology (Inchley et al., 2018). In total, 71,119 adolescents from 14 PCCE were recruited using the cluster sampling method, with classes or schools serving as primary sampling units. Adolescents completed a standardized self-administered questionnaire in classrooms. Relevant ethics committees in each participating country approved the surveys, complying with the HBSC study guidelines for the protection of human subjects concerning their safety and privacy (Inchley et al., 2018). Study participants and their schools, parents/guardians were fully informed about the research. Informed consent was obtained before the study (Inchley et al., 2018). This study was based on a publicly available anonymized database (The HBSC Data Management Centre, 2017). Details on the HBSC study design have been published elsewhere (Inchley et al., 2018).

### 2.2 Measures

#### 2.2.1 Bullying perpetration

Students were presented with a definition of bullying based on the Olweus bullying questionnaire (Olweus, 1996): “Here are some questions about bullying. We say a person is being bullied when another person or a group of people, repeatedly say or do unwanted nasty and unpleasant things to him or her. It also is bullying when a person is teased in a way he or she does not like or when he or she is left out of things on purpose. The person that bullies has more power than the person being bullied and wants to cause harm to him or her. It is not bullying when two people of about the same strength or power argue or fight.” After reading the definition, students answered a question on the frequency of bullying perpetration: 1) “How often have you taken part in bullying another person(s) at school in the past couple of months?” Five answer options were provided: 1) “I have not bullied another person(s) at school in the past couple of months”; 2) “It has happened once or twice”; 3) “2 or 3 times a month”; 4) “About once a week”; 5) Several times a week.” Based on the literature, responses were dichotomized into “at least 2–3 times” and “less than 2–3 times” (Inchley et al., 2020).

#### 2.2.2 Socioeconomic status

Adolescents reported their family socioeconomic status (SES) using the HBSC Family Affluence Scale (FAS III). Participants were asked: 1) “Does your family own a car, van or truck?” (no, one, two or more); 2) “Do you have your own bedroom for yourself?” (no, yes); 3) How many computers does your family own (including laptops and tablets, not including game consoles and smartphones) (none, one, two, more than two); 4) “How many bathrooms (room with a bath/shower or both) are in your home?” (none, one, two, more than two); 5) “Does your family have a dishwasher at home?” (no, yes); 6) “How many times did you and your family travel out of the country for a holiday/vacation last year?” (not at all, once, twice, more than twice) (Inchley et al., 2018). The responses were summed with higher scores indicating higher family SES. Then, adolescents were classified into low (20 %), middle (60 %), and high (20 %) SES groups using country-specific FAS III scores (Inchley et al., 2018).

#### 2.2.3 Country-level variables

The country estimates for economic development (GDP per capita) and income inequality (Gini index) were obtained from the 2017 World Bank statistics (World Bank., 2017a, World Bank., 2017b). A higher GDP per capita, and Gini index indicated greater economic development, and wider income inequality, respectively. All measures were centered at their means.

#### 2.2.4 Co-variates

Adolescents’ age, sex, and bullying victimization (at least 2–3 times) were included in the models as co-variates as they potentially affect bullying perpetration. Bullying victimization was measured by the question: “How often have you been bullied at school in the past couple of months?” with the same answer options as for bullying perpetration (see above) (Inchley et al., 2018).

### 2.3 Statistical analysis

**Fig. 1:**
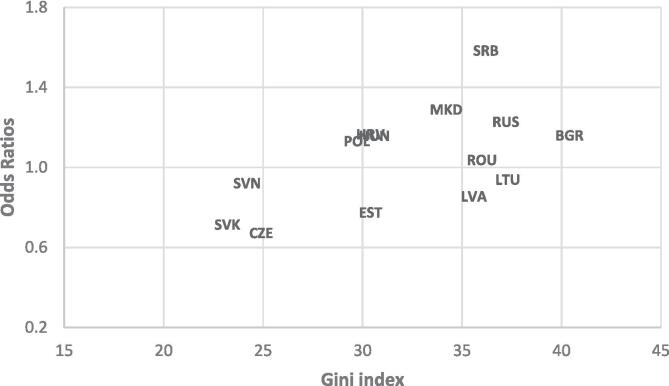
Odds ratios of bullying perpetration by family socioeconomic status (high 20% vs. low 20%) among 11 to 15-year-old adolescents. Notes: a higher Gini index denotes greater income inequality. BGR, Bulgaria; CZE, Czechia; EST, Estonia; HRV, Croatia; HUN, Hungary; LTU, Lithuania; LVA, Latvia; MKD, North Macedonia; POL, Poland; ROU, Romania; RUS, Russia; SRB, Serbia; SVN, Slovenia; SVK, Slovakia; (HRV and HUN are overlapping in the chart). a 1.5-column fitting image.

Multilevel logistic regression models were fitted with adolescents (level 1) nested into countries (level 2) using generalized linear mixed models (Laplace approximation) (Pinheiro and Chao, 2006). The estimates of random effect variances for schools and classes were low (ICC < 0.05) and were not included in the models. The random intercept model (Model 0) estimated the variance in bullying perpetration at the country level. Model 1 included individual-level variables (age, sex, SES, and bullying victimization). In Model 2, we assessed the effect of economic development (GDP per capita) on bullying perpetration. Next, we estimated the country differences in bullying perpetration attributable to income inequality (Models 3). A cross-level interaction between the Gini index and SES was assessed by introducing a random slope and creating a product term of those variables (Model 4). Weighted logistic regression analysis was used to estimate odds ratios of bullying perpetration comparing high- and low-SES adolescents across PCCE (Fig. 1). In a sensitivity analysis, we examined the potential for effect modification by age, sex, and GDP per capita of the above-mentioned relationships. A complete-case analysis method was used. *P*-values less than 0.05 indicated statistical significance. The intraclass correlation coefficient (ICC) was calculated by dividing the country-level variance V_C_ by the total variance using the linear threshold model (ICC = V_C_/[V_C_ + 3.29]) (Snijders and Bosker, 2011). Data were analyzed in R statistical software (v. 4.2.3, R Core Team [2023], R Foundation for Statistical Computing, Vienna, Austria. URL https://www.R-project.org/.)

## 3 Results

This study included 14 PCCE, with mean GDP per capita of USD 14,176 (range = 5,450–23,514) and a mean Gini index of 32.2 (range = 23.2–40.4). More details about the country socioeconomic characteristics can be found in Table 1.

**Table 1:** The number of participants and economic characteristics of 14 post-Communist countries of Europe participating in the 2017–18 Health Behavior in School-aged Children survey.

**Factors**	n = 71,119	GDP per capita (USD)	Gini index
Bulgaria	4548	8,366	40.4
Croatia	5169	13,629	30.4
Czechia	11,564	20,636	24.9
Estonia	4725	20,438	30.4
Hungary	3789	14,624	30.6
Latvia	4412	15,695	35.6
Lithuania	3797	16,885	37.3
North Macedonia	4658	5,450	34.2
Poland	5224	13,865	29.7
Romania	4567	10,807	36.0
Russia	4281	10,720	37.2
Serbia	3933	6,293	36.2
Slovakia	4785	17,538	23.2
Slovenia	5667	23,514	24.2

GDP, gross domestic product; note: a higher Gini index denotes greater income inequality.

Overall 71,119 adolescents participated in the survey. Almost half of the participants were males (49.7 %), with a mean age of 13.6 years (standard deviation = 1.7). Boys (odds ratio [OR] = 1.84; 95 % confidence interval [CI] = 1.74–1.95) and older adolescents (OR = 1.36; 95 % CI = 1.26–1.46) were more likely to bully others (see Table 2). Low- and high-SES adolescents had a 10–15 % greater risk (*P* < 0.05) of bullying perpetration compared to their peers from middle-SES families. Bullying victimization was associated with 5.77 times (95 % CI = 5.42–6.15) greater odds of bullying perpetration.

**Table 2:** Adjusted odds ratios (95% confidence intervals) and measures of country-level variance of bullying perpetration by individual and country-level characteristics among 11 to 15-year-old adolescents from 14 post-Communist countries of Europe participating in the 2017–18 Health Behavior in School-aged Children survey.

	**Model 0**	**Model 1**	**Model 2**	**Model 3**	**Model 4**
**Bullying perpetration (n = 5,755)**					
**Individual variables**					
Age (13 vs. 11 years)	–	**1.29 (1.20**–**1.39)**	**1.29 (1.20**–**1.39)**	**1.29 (1.20**–**1.39)**	**1.29 (1.21**–**1.39)**
Age (15 vs. 11 years)	–	**1.36 (1.26**–**1.46)**	**1.36 (1.26**–**1.46)**	**1.36 (1.26**–**1.46)**	**1.36 (1.26**–**1.46)**
Sex (boys vs. girls)	–	**1.84 (1.74**–**1.95)**	**1.84 (1.74**–**1.95)**	**1.84 (1.74**–**1.95)**	**1.84 (1.74**–**1.95)**
SES (middle vs. low)	–	**0.90 (0.84**–**0.97)**	**0.90 (0.84**–**0.97)**	**0.90 (0.84**–**0.97)**	**0.88 (0.81**–**0.95)**
SES (high vs. low)	–	1.05 (0.96–1.15)	1.05 (0.96–1.15)	1.05 (0.96–1.15)	1.02 (0.92–1.13)
Bullying victimization	–	**5.77 (5.42**–**6.15)**	**5.77 (5.42**–**6.15)**	**5.76 (5.41**–**6.14)**	**5.76 (5.41**–**6.14)**
**Country variables**					
GDP per capita	–	–	0.99 (0.95–1.03)	1.04 (0.99–1.08)	1.02 (0.98–1.07)
Gini index	–	–	–	**1.07 (1.03**–**1.12)**	1.04 (0.99–1.09)
**Cross-level interaction**					
Gini index*middle SES	–	–	–	–	**1.02 (1.01**–**1.04)^a^**
Gini index*high SES	–	–	–	–	**1.03 (1.01**–**1.05)^a^**
**Random effects**					
ICC	0.0644	0.0448	0.0437	0.0259	0.0341
PCV	–	30.3 %	32.0 %	59.7 %	47.0 %

SES, socioeconomic status; GDP, gross domestic product; ICC, intraclass correlation; PCV, proportional change in variance

note: a higher Gini index denotes greater income inequality.

a: The interaction term is more than 1.00, which indicates a synergistic effect between the Gini index and SES.

Bold values denote statistical significance (*P* < 0.05)

No statistically significant relationship (*P* > 0.05) was found between GDP per capita and bullying perpetration (Model 2). The contribution of GDP per capita to the total effect was minimal (32.0 % vs. 30.3 %). Wider income inequality (Gini index) increased the risk of bullying perpetration. About 27.7 % (59.7 % vs. 32.0 %) of country differences in bullying perpetration were attributable to large income inequality (Model 3). In cross-level interactions (Model 4), the risk of bullying preparation was the highest among high-SES adolescents from countries with wider income inequality (*P*-interaction = 0.003). Fig. 1 presents the odds ratios of bullying perpetration comparing high- vs. low-SES adolescents, which ranged from 0.67 in the least unequal (Czechia) to 1.59 in the most unequal countries (Serbia). In the sensitivity analyses, we did not observe substantial differences (*P*-interaction > 0.05) in the above-mentioned relationships between Gini index, family SES and bullying perpetration when comparing different age and sex groups, and GDP per capita.

## 4 Discussion

In this nationally representative study, we investigated the impact of individual and country socioeconomic characteristics on adolescent bullying perpetration in 14 PCCE with GDP per capita above USD 5,000. Both low- and high-SES adolescents were at greater risk of bullying perpetration than middle-SES adolescents. GDP per capita did not significantly explain the country differences in bullying perpetration. But, in line with our hypothesis, we found that large income inequality was a risk factor for bullying perpetration. These findings are consistent with Wilkinson’s hypothesis (Wilkinson and Pickett, 2011) that greater income inequality can increase school bullying due to increased social comparisons and status anxiety.

However, we also found that adolescents from high socioeconomic groups were more often involved in bullying perpetration in countries with high income inequality. Neo-liberal reforms and, as a result, the emergence of nouveaux-riches (new riches) in PCCE might provide important context to the above-mentioned relationship between high SES and bullying perpetration in countries with wide income inequality. A strong desire for status and recognition as members of a new social class and a struggle with distinguishing themselves from others due to their limited means beyond material possessions (Sampson, 1994, Ruegg, 2022) could have possibly made nouveaux-riches and their children more sensitive and vigilant to status loss. Therefore, in the face of actual or perceived threats to their high status, many wealthier adolescents, the so-called “golden youth” (Baker et al., 2007, Schimpfössl, 2018), might bully others to prevent status loss in their social groups. In addition, it is possible that the status-driven “golden youth” might try to gain superiority over those at the bottom of the socioeconomic hierarchy, demonstrating/reinforcing their high status (Volk et al., 2016).

Coburn suggests (Coburn, 2004, Coburn, 2000) that neoliberal policies create a greater power imbalance between higher and lower socioeconomic groups due to the decline in the welfare states and the weakening of institutions protecting the working class. Notably, a recent cross-national study has revealed that workplace bullying by managers might be more acceptable in PCCE (Power et al., 2013). Therefore, it is possible that in more neoliberal/unequal PCCE, bullying may also be viewed as a more socially acceptable way of hierarchy formation among adolescents. Favorable socioeconomic backgrounds might provide high-SES adolescents, particularly the “golden youth”, with greater resources to bully others and gain higher status among their peers. Further studies are needed to explore individual-level characteristics responsible for bullying perpetration among high-SES adolescents from PCCE with wide income inequality.

Our study had several strengths. First, HBSC is a WHO collaborative study employing standardized methodology and validated tools. Second, it is one of the largest and nationally representative surveys conducted in PCCE. Third, the proportion of missing values was low (5 % or less) without regular patterns, and it is unlikely that missing values introduced bias into our results. Finally, we used multilevel models that accounted for the hierarchical nature of the data. Nevertheless, this study also has limitations. Self-reported data on bullying might be subject to desirability bias, especially among high-SES adolescents. Therefore, the overall prevalence of bullying perpetration and the magnitude of the associations might be underestimated. The ecological nature of the country variables requires further studies to identify the exact causal mechanisms for the observed relationships. It is worth noting that the number of level 2 units was relatively small (14), which could have potentially affected our ability to detect differences related to country-level variables (type 2 error). Also, including additional countries in further research would minimize the risk of getting false positive results (type 1 error).

## 5 Conclusion

In conclusion, our findings suggest that high income inequality might contribute to school bullying among adolescents in PCCE, particularly those from high SES families. The necessary political and economic interventions should focus on improving the country socioeconomic environment in PCCE. Moving towards less neoliberal policies and creating a strong welfare state that promotes the economic and social well-being of the population and reduces income inequality might be a necessary component of macro-level strategies aimed at preventing bullying among adolescents. Targeted public health interventions might be required for high-SES adolescents in countries with wide income inequality.

## Funding

This research did not receive any specific grant from funding agencies in the public, commercial, or not-for-profit sectors.

## CRediT authorship contribution statement

**Armen Albert Torchyan:** Conceptualization, Methodology, Formal analysis, Data curation, Visualization, Writing – original draft. **Inge Houkes:** Conceptualization, Methodology, Supervision, Visualization, Writing – review & editing. **Hans Bosma:** Conceptualization, Methodology, Supervision, Visualization, Writing – review & editing.

## Declaration of competing interest

The authors declare that they have no known competing financial interests or personal relationships that could have appeared to influence the work reported in this paper.

## Data Availability

The data that support the findings of this study are available in the HBSC Data Management Centre at https://www.uib.no/en/hbscdata/113290/open-access
